# Supplementary material for: Telehealth in outpatient care for children and adolescents with chronic conditions during the COVID-19 pandemic: A scoping review protocol
Source: PLoS One. 2022 Jun 10;17(6):e0269821. doi: 10.1371/journal.pone.0269821 (PMC9187091; doi:10.1371/journal.pone.0269821)
Supplement: Attachment — Submitted filename: PONE-D-21-25612 Dated 25 Dec.pdf [file pone.0269821.s002.pdf]

PLOS ONE

Telehealth in outpatient care for children and adolescents with chronic conditions during the COVID-19 pandemic: a scoping review protocol

--Manuscript Draft--

**Manuscript Number:**	PONE-D-21-25612
**Article Type:**	Registered Report Protocol
**Full Title:**	Telehealth in outpatient care for children and adolescents with chronic conditions during the COVID-19 pandemic: a scoping review protocol
**Short Title:**	Telehealth for children and adolescents with chronic conditions in the COVID-19 pandemic
**Corresponding Author:**	REGINA APARECIDA GARCIA LIMA, PhD<br>Universidade de São Paulo Escola de Enfermagem de Ribeirão Preto<br>Ribeirao Preto, SP BRAZIL
**Keywords:**	Telehealth; Outpatient care; children; adolescents; Chronic conditions; COVID-19
**Abstract:**	Introduction<br><br>Outpatient care for children and adolescents with chronic conditions needs to be continuous and programmed, encompassing comprehensive care, with periodically scheduled consultations, exams, and procedures, to promote quality of life and reduce mortality. In the context of the new coronavirus pandemic, however, outpatient care for children and adolescents with chronic conditions, in person, was hampered in favor of social isolation, a necessary sanitary measure to reduce and prevent the spread of Coronavirus Disease 2019. In response to this need, studies suggest telehealth in pediatrics as a fertile and expanding field especially in times of pandemics. Here, we aimed to map the evidence related to telehealth in outpatient care for children and adolescents with chronic conditions during the COVID-19 pandemic, to identify which strategies were implemented and their impacts on the continuity of care.<br><br>Methods<br><br>A scoping review protocol is reported, adhering to the PRISMA-P statement and guided by the Scoping Reviews Manual of the Joanna Briggs Institute. The search for evidence will cover the following databases: MEDLINE/PubMed, Cochrane Libary; Embase; Web of Science; Scopus; Cinahl and PsycINFO, plus additional sources, such as The British Library, Google Scholar, and Preprints [medRXiv]. No date or language restrictions will be employed in this scoping review. Two independent researchers will conduct the search strategy, study selection, data charting, and data synthesis.<br><br>Results<br><br>The findings will be presented through tables, charts, narrative summaries, and assessed based on the type of data charted as well as outcomes. Additionally, the meaning of these findings will be considered as they relate to the guiding question, the characterization and measurement of the impact of different telehealth modalities used in outpatient care for children and adolescents with chronic conditions during the COVID-19 pandemic, and the implications for practice and further research.<br><br>Discussion<br><br>To the best of our knowledge, this will be the first scoping review to look specifically at the telehealth modalities to be used in outpatient care for children and adolescents with chronic conditions during the COVID-19 pandemic. We expect that our results will be of interest to practitioners as well as researchers concerned with this particular emerging issue in the pandemic context. Also, the plans for the dissemination of this study comprise peer-reviewed publication and conference presentations.<br><br>Open Science Framework Registration

Powered by Editorial Manager® and ProduXion Manager® from Aries Systems Corporation

	osf.io/5pqgu
**Order of Authors:**	Luís Carlos Lopes-Junior, Ph.D
	Larissa Karoline Dias da Silva Casemiro
	Fabrine Aguilar Jardim
	Mariane Caetano Sulino
	Regina Aparecida Garcia Lima
**Additional Information:**	
**Question**	**Response**
**Financial Disclosure**<br><br>Enter a financial disclosure statement that describes the sources of funding for the work included in this submission. Review the submission guidelines for detailed requirements. View published research articles from *PLOS ONE* for specific examples.<br><br>This statement is required for submission and **will appear in the published article** if the submission is accepted. Please make sure it is accurate.<br><br>**Unfunded studies**<br>Enter: *The author(s) received no specific funding for this work.*<br><br>**Funded studies**<br>Enter a statement with the following details:<br>• Initials of the authors who received each award<br>• Grant numbers awarded to each author<br>• The full name of each funder<br>• URL of each funder website<br>• Have or will the sponsors or funders play any role in the study design, data collection (when applicable) and analysis, decision to publish, or preparation of the manuscript?<br>• **NO** - Include this sentence at the end of your statement: *The funders had and will not have a role in study design, data collection and analysis, decision to publish, or preparation of the manuscript.*<br>• **YES** - Specify the role(s) played.<br><br>* typeset	This study protocol is supported by the CAPES Higher Education Improvement Coordination, Notice: Emergency Action/COVID-19. Process Number: 88887.508473/2020-00. Doctoral scholarship to the first author (LKDSC).<br><br>The funders had and will not have a role in study design, data collection and analysis, decision to publish, or preparation of the manuscript.
**Competing Interests**<br><br>Use the instructions below to enter a	The authors have declared that no competing interests exist.

competing interest statement for this submission. On behalf of all authors, disclose any competing interests that could be perceived to bias this work—acknowledging all financial support and any other relevant financial or non-financial competing interests.<br><br>This statement is required for submission and **will appear in the published article** if the submission is accepted. Please make sure it is accurate and that any funding sources listed in your Funding Information later in the submission form are also declared in your Financial Disclosure statement.<br><br>View published research articles from *PLOS ONE* for specific examples.<br><br>**NO authors have competing interests**<br><br>Enter: *The authors have declared that no competing interests exist.*<br><br>**Authors with competing interests**<br><br>Enter competing interest details beginning with this statement:<br><br>*I have read the journal's policy and the authors of this manuscript have the following competing interests: [insert competing interests here]*<br><br>* typeset	
**Ethics Statement**<br><br>Enter an ethics statement for this submission. This statement is required if the study involved:<br><br>• Human participants<br>• Human specimens or tissue<br>• Vertebrate animals or cephalopods<br>• Vertebrate embryos or tissues<br>• Field research	Not applicable

Write "N/A" if the submission does not require an ethics statement.

General guidance is provided below. Consult the submission guidelines for detailed instructions. **Make sure that all information entered here is included in the Methods section of the manuscript.**

Format for specific study types

Human Subject Research (involving human participants and/or tissue)

- Give the name of the institutional review board or ethics committee that approved the study
- Include the approval number and/or a statement indicating approval of this research
- Indicate the form of consent obtained (written/oral) or the reason that consent was not obtained (e.g. the data were analyzed anonymously)

Animal Research (involving vertebrate animals, embryos or tissues)

- Provide the name of the Institutional Animal Care and Use Committee (IACUC) or other relevant ethics board that reviewed the study protocol, and indicate whether they approved this research or granted a formal waiver of ethical approval
- Include an approval number if one was obtained
- If the study involved *non-human primates*, add *additional details* about animal welfare and steps taken to ameliorate suffering
- If anesthesia, euthanasia, or any kind of animal sacrifice is part of the study, include briefly which substances and/or methods were applied

Field Research

Include the following details if this study involves the collection of plant, animal, or other materials from a natural setting:

- Field permit number
- Name of the institution or relevant body that granted permission

Powered by Editorial Manager® and ProduXion Manager® from Aries Systems Corporation

**Data Availability**<br><br>Authors are required to make the data underlying their research findings fully available, without restriction.<br><br>If the manuscript reports pilot results, the data underlying those results should be made available at the time of publication. Submission of this Registered Report Protocol implies a commitment that the data collected in the study, upon completion, will be made available with the Registered Report publication.<br><br>When legal or ethical requirements prohibit public data sharing, authors must indicate how researchers may obtain access to the data. See the PLOS Data Policy and FAQ for detailed information.<br><br>A Data Availability Statement describing where the data can be found is required at submission. Your answers to this question constitute the Data Availability Statement and **will be published in the article**, if accepted.<br><br>**Important:** Stating 'data available on request from the author' is not sufficient. If your data are only available upon request, select 'No' for the first question and explain your situation in the text box.<br><br>Do the authors confirm that all data underlying the findings of the study outlined in this Registered Report Protocol will be made fully available without restriction?	Yes - any pilot data reported in this submission are fully available and data collected during the study will be made fully available without restriction upon study completion
**Describe where the data may be found in full sentences. If you are copying our sample text, replace any instances of XXX with the appropriate details.**<br><br>• If **no pilot data are reported in the**	All relevant data from this study will be made available upon study completion.

**manuscript**, please enter the following: *All relevant data from this study will be made available upon study completion.*<br>• If the manuscript reports pilot data **held in a public repository**, include URLs, accession numbers or DOIs. If this information will only be available after acceptance, indicate this by ticking the box below. For example: *All XXX files are available from the XXX database (accession number(s) XXX, XXX.).*<br>• If the manuscript reports pilot data contained **within the manuscript and/or Supporting Information files**, enter the following: *All relevant data are within the manuscript and its Supporting Information files.*<br>• If neither of these applies but you are able to provide **details of access elsewhere**, with or without limitations, please do so. For example:<br><br>*Data cannot be shared publicly because of [XXX]. Data are available from the XXX Institutional Data Access / Ethics Committee (contact via XXX) for researchers who meet the criteria for access to confidential data.*<br><br>*The data underlying the results presented in the study are available from (include the name of the third party and contact information or URL).*<br>• This text is appropriate if the data are owned by a third party and authors do not have permission to share the data.<br><br>* typeset	
Additional data availability information:	Tick here if the URLs/accession numbers/DOIs will be available only after acceptance of the manuscript for publication so that we can ensure their inclusion before publication.

main document Click here to access/download;Manuscript;Main document final 9.8.2021.docx

PLOS One

Full title: **Telehealth in outpatient care for children and adolescents with chronic conditions during the COVID-19 pandemic: a scoping review protocol**

Short Title: **Telehealth for children and adolescents with chronic conditions in the COVID-19 pandemic**

Authors:

Larissa Karoline Dias da Silva Casemiro[1*], RN, MSc., Ph.D. student. E-mail: larissakarolineds@gmail.com
Luís Carlos Lopes-Júnior[2*], RN, Ph.D. E-mail: lopesjr.lc@gmail.com
Fabrine Aguilar Jardim[1], RN, MSc., Ph.D. student. E-mail: fabrineaguilar@gmail.com
Mariane Caetano Sulino[1], RN, MSc., Ph.D. student. E-mail: mariane.caetano.sulino@gmail.com
Regina Aparecida Garcia de Lima[1#], RN, Ph.D. E-mail: limare@eerp.usp.br

***Master Larissa Karoline Dias da Silva Casemiro (LKDSC) and** *Dr. Luís Carlos Lopes-Júnior (LCLJ) collaborated equally on this work and are joint first authors*

Affiliations:
[1]University of São Paulo at Ribeirão Preto College of Nursing, Ribeirão Preto, SP. Brazil.
[2]Health Sciences Center at the Federal University of Espírito Santo (UFES). Vitoria, ES, Brazil.

#Corresponding author: Regina Aparecida Garcia de Lima. RN., Ph.D. Full Professor, Department of Maternal-Infant and Public Health Nursing, Ribeirão Preto College of Nursing – PAHO/WHO Collaborating Centre for Nursing Research Development, University of São Paulo, Brazil E-mail: limare@eerp.usp.br; Phone: +5516 3315-0542. ORCID: 0000-0002-0611- 562

Author Contributions
Conceptualization: LKDSC and RAGL
Methodology: LCLJ
Resources: RAGL
Acquisition/interpretation of data for the work: LKDSC, FJA, MCS, LCLJ.
Supervision: LCLJ and RAGL
Writing – original draft: LKDSC, FJA, MCS, LCLJ and RAGL
Writing – review & editing, and approving the final version: LKDSC, FJA, MCS, LCLJ and RAGL
Guarantors: LKDSC and RAGL
Competing interests: None declared
Acknowledgements: None to declare.

Funding: This study protocol is supported by the CAPES Higher Education Improvement Coordination, Notice: Emergency Action/COVID-19. Process Number: 88887.508473/2020-00.

Patient consent for publication: Not required

Word Count: 2050 words.
Disclaimer: The views of the authors do not necessarily reflect those of the NHS, NIHR or the Department of Health.
Sponsorship: None.
Patient consent: Not required.
Provenance and peer review: Not commissioned; externally peer reviewed.

ABSTRACT

Introduction: Outpatient care for children and adolescents with chronic conditions needs to be continuous and programmed, encompassing comprehensive care, with periodically scheduled consultations, exams, and procedures, to promote quality of life and reduce mortality. In the context of the new coronavirus pandemic, however, outpatient care for children and adolescents with chronic conditions, in person, was hampered in favor of social isolation, a necessary sanitary measure to reduce and prevent the spread of Coronavirus Disease 2019. In response to this need, studies suggest telehealth in pediatrics as a fertile and expanding field especially in times of pandemics. Here, we aimed to map the evidence related to telehealth in outpatient care for children and adolescents with chronic conditions during the COVID-19 pandemic, to identify which strategies were implemented and their impacts on the continuity of care.

Methods: A scoping review protocol is reported, adhering to the PRISMA-P statement and guided by the Scoping Reviews Manual of the Joanna Briggs Institute. The search for evidence will cover the following databases: MEDLINE/PubMed, Cochrane Libary; Embase; Web of Science; Scopus; Cinahl and PsycINFO, plus additional sources, such as The British Library, Google Scholar, and Preprints [medRXiv]. No date or language restrictions will be employed in this scoping review. Two independent researchers will conduct the search strategy, study selection, data charting, and data synthesis.

Results: The findings will be presented through tables, charts, narrative summaries, and assessed based on the type of data charted as well as outcomes. Additionally, the meaning of these findings will be considered as they relate to the guiding question, the characterization and measurement of the impact of different telehealth modalities used in outpatient care for children and adolescents with chronic conditions during the COVID-19 pandemic, and the implications for practice and further research.

Discussion: To the best of our knowledge, this will be the first scoping review to look specifically at the telehealth modalities to be used in outpatient care for children and adolescents with chronic conditions during the COVID-19 pandemic. We expect that our results will be of interest to practitioners as well as researchers concerned with this particular emerging issue in the pandemic context. Also, the plans for the dissemination of this study comprise peer-reviewed publication and conference presentations.

Open Science Framework Registration: osf.io/5pqgu

Keywords: Telehealth; Outpatient care; Children; Adolescents; Chronic conditions; COVID-19.

Introduction

Chronic conditions encompass a broad category of health problems with biological, psychological, or cognitive causes [1]. These include transmissible diseases such as HIV/AIDS and tuberculosis; non-communicable diseases such as cardiovascular disease, cancer, chronic respiratory diseases, diabetes; long-term mental disorders, and structural disabilities [2].

The prevalence of chronic conditions in childhood has increased by more than 400% in the last 50 years, favoring the occurrence of comorbidities, a large number of hospitalizations, and a high degree of mortality [3]. A retrospective observational stud in a pediatric intensive care unit of a hospital in London found that 46% of hospitalized patients had at least one chronic health condition. Also, these children remained hospitalized longer, increasing the chances of mortality [4].

A prospective, multicenter, observational cohort study conducted in 19 pediatric care units of hospitals in Argentina identified that 48% of hospitalized children had one or more chronic conditions, the most prevalent being cardiovascular diseases (22.24%), neuromuscular diseases (18.75%), and genetic diseases (17.7%). The results also showed that children with chronic conditions had a greater need for tracheostomy and mechanical ventilation when compared to children hospitalized for acute conditions, which increased the morbidity and complexity of care [5].

Due to clinical weaknesses, children and adolescents with chronic conditions demand high hospital readmission rates, as exemplified in a cohort study in 37 pediatric hospitals in the USA with a sample of 69,294 children. Of the total, 18.8% were readmitted to the service, 89.0% of whom had complex chronic conditions, 52.6% used technological devices, and 2.9% were readmitted more than four times within a year [6].

In this sense, outpatient care for children and adolescents with chronic conditions needs to be continuous and programmed, encompassing comprehensive care, with periodically scheduled consultations, exams, and procedures [1], to promote quality of life and reduce mortality. In the context of the new coronavirus pandemic, however, outpatient care for children and adolescents with chronic conditions, in person, was hampered [7] in favor of social isolation, a necessary sanitary measure to reduce and prevent the spread of Coronavirus Disease 2019 (COVID-19) [8].

Research has shown that COVID-19 can lead to more severe complications in people with chronic conditions [9,10], which reinforces the recommendations of social isolation for this audience. Thus, it is necessary to reflect on innovative outpatient care models that contribute to the continuity of care and comprehensive health care for patients with chronic conditions.

In response to this need, studies suggest telehealth in pediatrics as a fertile and expanding field, especially in times of a pandemic [11–13]. According to the World Health Organization, telehealth refers to health services at a distance, synchronously or asynchronously, through

information and communication technologies. This modality makes it possible to carry out clinical care, diagnoses, treatments, prevention of diseases and injuries, research, and evaluation. Another potential use is in continuing education for health professionals [14]. Telehealth is being used in several countries worldwide, such as Brazil [15], Malaysia [16], England [17], Italy [18], Canada [19], United States of America [20], Argentina [21], to nullify the barriers imposed by the COVID-19 pandemic.

Telehealth can positively affect the care of children and adolescents with chronic conditions. They need long-term, programmed care, but traveling to the health service can lead to a greater risk of contagion. Therefore, innovative care models are needed, such as care provision using remote methods. Hence, this study aims to map the evidence related to telehealth in outpatient care for children and adolescents with chronic conditions during the COVID-19 pandemic. Another aim is to identify the strategies implemented and their impacts on the continuity of care.

Methods

This study is a scoping review (ScR) type, which aims to map the key concepts of a particular field of research to clarify definitions and conceptual limits. Additional aims are to identify evidence, analyze knowledge gaps and examine how research is conducted in a given field [22], providing a descriptive analysis of the included studies [23].

The methodological framework described by Arksey and O'Malley [22], and further outlined in the Scoping Reviews Manual [24] of the Joanna Briggs Institute (JBI) serves as a guide for this ScR. The five steps of the ScR include (I) identifying the research question; (II) identifying the relevant studies; (III) selecting the studies; (IV) charting the data; and (V) collating, summarizing, and reporting the results; and will be depicted as follows. Additionally, this protocol has been registered within the Open Science Framework (osf.io/5pqgu) and will be reported in line with the Preferred Reporting Items for Systematic Reviews and Meta-Analyses Protocols (PRISMA-P) statement [25]. Finally, this ScR will be reported following the Preferred Reporting Items for Systematic Review and Meta-Analyses extension for Scoping Reviews (PRISMA-ScR) [26].

Step 1: Identifying the research question

For the construction of the research question, the PCC (Population, Concept, and Context) framework was applied to identify the main concepts and is described in detail as follows:

Population

The target population of this scoping review will be children and adolescents aged 0-19 years with chronic conditions. The World Health Organization (WHO) defines a child as a person under 19 years of age, and this term includes newborns (0 and 28 days), babies (between 0 and 11 months), and adolescents (10 and 19 years) [27].

WHO defines chronic conditions as non-communicable diseases (e.g., diabetes, heart disease, lung diseases, and cancer), persistent communicable diseases (e.g., HIV/AIDS and tuberculosis), long-term mental disorders (e.g., schizophrenia and depression), and ongoing physical impairments or structural problems (e.g., amputations, blindness, and musculoskeletal disorders) [2].

Concept

This scoping review will include studies that address telehealth in child and adolescent care with chronic conditions during the COVID-19 pandemic due to the interruption of face-to-face care in outpatient clinics. For this study, telehealth, according to WHO, refers to the use of information and communication technologies for remote diagnosis and treatment of patients, aiming to promote individual and family health [14].

Context

The context analyzed in this scoping review is outpatient care, which occurs when the patient travels to a health environment to receive care but will not necessarily spend the night; thus, this review does not relate to care provided during hospitalizations or at home [28]. In this scoping review, we link outpatient care to COVID-19 - a disease caused by a new type of beta-coronavirus, capable of causing respiratory, enteric, liver, and neurological conditions [29]. COVID-19 was first identified in a group of patients with pneumonia in December 2019 in Wuhan, China [29], and WHO declared it a pandemic on March 11, 2020 [30].

Thus, after applying the PCC framework [22], the research question raised was: "What are the scientific productions available in the literature on the use of telehealth in outpatient care for children and adolescents with chronic conditions during the COVID-19 pandemic?"

Step 2: Identifying the relevant studies

The search for evidence will cover the following databases: Medical Literature Analysis and Retrieval System Online (MEDLINE) via PubMed, Cochrane Libary; Excerpta Medica database (Embase); Web of Science; Scopus; Cumulative Index to Nursing and Allied (Cinahl) and Psychology Information (PsycINFO), plus additional sources, such as The British Library (UK), Google Scholar, and Preprints [medRXiv]. For the search strategy, we will include a combination of subject headings, e.g., Medical Subject Headings (MeSH), Emtree, Cinahl headings, Thesaurus, using the Boolean terms AND/OR [31,32]. Additionally, the reference list of all included studies will be reviewed for additional relevant studies. No date or language restrictions will be employed in this scoping review.

To structure the search, as already mentioned, searches were carried out in MEDLINE/PubMed to find the best combination of MeSH terms, synonyms as well as free text words, which will be later adjusted for each electronic database. Following the PCC framework

(Population/Concept/Context), a pilot search strategy was established for MEDLINE/PubMed (Table 1):

Table 1 Preliminary pilot search strategy in MEDLINE/PubMed

MEDLINE/PubMed	**Search strategy**
	(P) - Population<br>**#1** ((“Infant” [MeSH Terms] OR “Infants” [All Fields] OR “Child, Preschool” [MeSH Terms] OR “Preschool Child” [All Fields] OR “Children, Preschool” [All Fields] OR “Preschool Children” [All Fields] OR “Child” [MeSH Terms] OR “Children” OR “Adolescent” [MeSH Terms] OR “Adolescents” [All Fields] OR “Adolescence” [All Fields] OR “Teens” [All Fields] OR “Teen” [All Fields] OR “Teenagers” [All Fields] OR “Teenager” [All Fields] OR “Youth” [All Fields] OR “Youths” [All Fields]))
	(C) - Concept<br>**#2** ((“Telemedicine” [MeSH Terms] OR “Mobile Health” [All Fields] OR “Health, Mobile” [All Fields] OR “mHealth” [All Fields] OR “Telehealth” [All Fields] OR “eHealth” [All Fields] OR “Telehealth” [All Fields] OR “Teleconsultation” [All Fields] OR “Telemonitoring” [All Fields] OR “Telenursing” [All Fields]))
	(C) - Context<br>**#3** (("COVID-19" [MeSH Terms] OR “COVID 19” [All Fields] OR “COVID-19 Virus Disease” [All Fields] OR “2019-nCoV Infection” [All Fields] OR “2019 nCoV Infection” [All Fields] OR “Coronavirus Disease-19” [All Fields] OR “Coronavirus Disease 19” [All Fields] OR “2019 Novel Coronavirus Disease” [All Fields] OR “2019 Novel Coronavirus Infection” [All Fields] OR “2019-nCoV Disease” [All Fields] OR “2019 nCoV Disease” [All Fields] OR “Coronavirus Disease 2019” [All Fields] OR “SARS Coronavirus 2 Infection” [All Fields] OR “SARS-CoV-2 Infection” [All Fields] OR “Infection, SARS-CoV-2” [All Fields] OR “SARS CoV 2 Infection” [All Fields] OR “SARS-CoV-2 Infections” [All Fields] OR “COVID-19 Pandemic” [All Fields] OR “COVID 19 Pandemic” [All Fields] OR “COVID-19 Pandemics” [All Fields] OR “Pandemic, COVID-19” [All Fields] OR “SARS-CoV-2" [MeSH Terms] OR “2019 Novel Coronavirus” OR “Coronavirus, 2019 Novel” OR “Novel Coronavirus, 2019” OR “SARS-CoV-2 Virus” OR “SARS CoV 2 Virus” OR “2019-nCoV” OR “COVID-19 Virus” OR “SARS Coronavirus 2” OR “Coronavirus 2, SARS” OR “Severe Acute Respiratory Syndrome Coronavirus” OR “SARS-CoV-2 variants” [Supplementary Concept] OR “SARS-CoV-2 B.1.1.7 variant” [All Fields]))
	**#4:** #1 AND #2 AND #3

Step 3: Selecting the studies

- *Inclusion criteria:* all the primary studies, experience reports, dissertations, and theses related to the use of telehealth in outpatient care for children and adolescents with chronic conditions during the COVID-19 pandemic will be included. It is noteworthy that there will be no date or language restriction for the study selection.
- *Exclusion criteria:* studies with adults, even if children and adolescents participate; and studies on acute illnesses that address chronic diseases will be excluded.

After searching for studies, articles will be downloaded to the Endnote Web™ bibliographic software to store, organize, manage all references and identify duplicates. The studies will then be exported to the Rayyan™ application, which assists in the screening and selection of studies. At this stage, the titles and abstracts will be read to carry out an initial assessment of the evidence. Two independent researchers (LKDSC and LCLJ) will search and screen the records by titles and abstract into the Rayyan™ app. After the initial screening, the same two independent researchers

(LKDSC and LCLJ) will assess the full text of studies retrieved for inclusion/exclusion, using the Rayyan™ app. A third reviewer (RAGL) will decide on any disagreements as to the selected studies.

Step 4: Charting the data

Two reviewers/authors (LKDSC and LCLJ) will independently chart the data for each included study based on previously published forms [23, 32-36]. The expected date of completion of this scoping review will be December 2021. Information to be extracted includes a) identification of the study and objectives; b) study population and baseline characteristics; c) type of exposure; d) study method; e) recruitment methods; f) times of measurement; g) follow-up; h) outcomes; i) main findings; j) clinical and epidemiological significance; and k) conclusions [23, 32-36].

Results

Step 5: Collating, summarizing, and reporting the results

A flowchart diagram will describe the entire study selection process [23]. For data analysis, a thematic evaluation will be done to describe the mapping and telehealth strategies and modalities used in outpatient care for children and adolescents with chronic conditions during the COVID-19 pandemic, with a view to reporting which actions were implemented and their impacts on the continuity of care. The systematic assessment of study quality (risk of bias of the included studies) will not be performed, as the intent of this ScR is to provide the mapping and extension of the literature rather than a critical appraisal of the studies like in systematic reviews [23]. Our findings will be presented through tables, charts, narrative summaries, and will be assessed based on the type of data charted and the outcomes. Additionally, the meaning of these findings will be considered as they relate to the guiding question, the characterization and measurement of the impact of different telehealth modalities used in outpatient care for children and adolescents with chronic conditions during the COVID-19 pandemic, and the implications for practice and further research.

Discussion

The purpose of this ScR is to map the evidence related to telehealth in outpatient care for children and adolescents with chronic conditions during the COVID-19. In addition, the purpose is to identify which strategies were implemented and their impacts on the continuity of care. Thus, we will undertake an ScR. This includes a comprehensive literature search without restrictions to language or date of publication. We will search across multiple electronic databases, additional sources, and grey literature. The ScR method serves to chart and map results and to establish the directions for future research.

It is noteworthy that there are two aspects in which the study within this protocol differs from others. Firstly, the ScR is more suitable for our study theme than a systematic review. Our research question is quite broad and focuses on mapping the extension of the evidence available. Secondly, we combine qualitative and quantitative methods to report our results, using thematic themes and bibliometric trends to provide efficient guidance and meaningful insights into this field.

The use of new and varied methods to review the evidence and collate and summarize our findings represents the strength of our study. To the best of our knowledge, this will be the first ScR that combines these methods to look specifically at the telehealth modalities used in outpatient care for children and adolescents with chronic conditions during the COVID-19 pandemic.

The limitations in this study are related to the quality of the records scrutinized in this ScR. We expect that our results will be of interest to practitioners, researchers, and anyone concerned with this particular emerging issue in the pandemic context. In addition, the plans of dissemination of this study comprise peer-reviewed publication and conference presentations.

References

1. Brasil. Política nacional de atenção integral à saúde da criança: orientação para a implementação [Internet]. Brasília: Ministério da Saúde; 2018. 180 p. Available from: http://www.saude.pr.gov.br/arquivos/File/Politica_Nacional_de_Atencao_Integral_a_Saude_da_Crianca_PNAISC.pdf.

2. World Health Organization (WHO). Innovative Care for Chronic Conditions: Building Blocks for Action. Geneva: World Health Organization (WHO/MNC/CCH/02.01). Geneva World Heal Organ. 2002.

3. Cohen E, Berry JG, Sanders L, Schor EL, Wise PH. Status Complexicus? The Emergence of Pediatric Complex Care. Pediatrics. 2018 Mar;141(Suppl 3):S202-S211. https://doi.org/10.1542/peds.2017-1284E. PMID: 29496971.

4. O'Brien S, Nadel S, Almossawi O, Inwald DP. The Impact of Chronic Health Conditions on Length of Stay and Mortality in a General PICU. Pediatr Crit Care Med. 2017 Jan;18(1):1-7. https://doi.org/10.1097/PCC.0000000000000976. PMID: 27811531.

5. Arias López MDP, Fernández AL, Fiquepron K, Meregalli C, Ratto ME, Siaba Serrate A; SATI-Q Pediatric Group. Prevalence of Children With Complex Chronic Conditions in PICUs of Argentina: A Prospective Multicenter Study. Pediatr Crit Care Med. 2020;21(3):e143-e151. https://doi.org/10.1097/PCC.0000000000002223. PMID: 31851126.

6. Berry JG, Hall DE, Kuo DZ, Cohen E, Agrawal R, Feudtner C, Hall M, Kueser J, Kaplan W, Neff J. Hospital utilization and characteristics of patients experiencing recurrent readmissions within children's hospitals. JAMA. 2011 ;305(7):682-90. https://doi.org/10.1001/jama.2011.122. PMID: 21325184.

7. Seth R. COVID-19 pandemic: The challenges for pediatric oncology. Indian Pediatrics. 2020;57(6):589.

8. Schuchmann AZ, Schnorrenberger BL, Chiquetti ME, Gaiki RS, Raimann BW, Maeyama MA. Vertical social isolation X Horizontal social isolation: health and social dilemas in copping with the COVID-19 pandemic. Brazilian J Heal Rev. 2020;3(2):3556–76. https://doi.org/10.34119/bjhrv3n2-185

9. Antunes BS, Kinalski DDF, Schneider V, Silva AF da, Motta M da GC da. COVID-19 and children with chronic diseases: scope review. RSD 2020;9(12):e5391210748. https://doi.org/10.33448/rsd-v9i12.10748.

10. Kompaniyets L, Agathis NT, Nelson JM, Preston LE, Ko JY, Belay B, et al. Underlying Medical Conditions Associated With Severe COVID-19 Illness Among Children. JAMA Netw Open. 2021;4(6):e2111182. https://doi.org/10.1001/jamanetworkopen.2021.11182. PMID: 34097050.

11. Puthenpura V, Du N, Hauptman L, Porto AF. Disparities in Telehealth Utilization Within Pediatric Gastroenterology: One Academic Center's Experience. J Pediatr Gastroenterol Nutr. 2021. https://doi.org/10.1097/MPG.0000000000003167. PMID: 34016884.

12. Howie F, Kreofsky BL, Ravi A, Lokken T, Hoff MD, Fang JL. Rapid Rise of Pediatric Telehealth During COVID-19 in a Large Multispecialty Health System. Telemed J E Health. 2021. https://doi.org/10.1089/tmj.2020.0562. PMID: 33999718.

13. Sattar S, Kuperman R. Telehealth in pediatric epilepsy care: A rapid transition during the COVID-19 pandemic. 2020;111:107282. https://doi.org/10.1016/j.yebeh.2020.107282. PMID: 32759065.

14. World Health Organization. WHO Guideline on Health Workforce Development, Attraction, Recruitment and Retention in Rural and Remote Areas. 2021. 1–104 p.

15. Brasil. Ministério da Saúde. Portaria nº 467, de 20 de março de 2020. Dispõe, em caráter excepcional e temporário, sobre as ações de Telemedicina, com o objetivo de regulamentar e operacionalizar as medidas de enfrentamento da emergência de saúde pública de importância internacional previstas no art. 3º da Lei nº 13.979, de 6 de fevereiro de 2020, decorrente da epidemia de COVID-19. Brasil, 2020.

16. Malaysian Medical Council. MMC's Advisory on Virtual Consultation (during the Covid19 pandemic). 2020;1971(Amended). Available from: https://fmrac.ca/fmrac.

17. Rajgopal A, Li CR, Shah S, Sundar Budhathoki S. The use of telehealth to overcome barriers to mental health services faced by young people from Afro-Caribbean backgrounds in England during the COVID-19 pandemic. J Glob Health. 2021 Mar 1;11:03040. https://doi.org/10.7189/jogh.11.03040. PMID: 33880177.

18. Provenzi L, Grumi S, Gardani A, Aramini V, Dargenio E, Naboni C, et al. Italian parents welcomed a telehealth family-centred rehabilitation programme for children with disability during COVID-19 lockdown. Acta Paediatr Int J Paediatr. 2021;110(1):194–6. https://doi.org/10.1111/apa.15636. PMID: 33098116.

19. Qiu Y, Coulson S, McIntyre CW, Wile B, Filler G. Adolescent and caregiver attitudes towards telemedicine use in pediatric nephrology. BMC Health Serv Res. 2021 Jun 1;21(1):537. https://doi.org/10.1186/s12913-021-06506-0. PMID: 34074281.

20. Van Houten L, Deegan K, Siemer M, Walsh S. A telehealth initiative to decrease no-show rates in a pediatric asthma mobile clinic. J Pediatr Nurs. 2021;59:143–50.

https://doi.org/10.1016/j.pedn.2021.04.005. PMID: 33887684.

21. Fortini S, Espeche A, Caraballo R. Telemedicine and epilepsy: A patient satisfaction survey of a pediatric remote care program. Epilepsy Res. 2020;165:106370. https://doi.org/10.1016/j.eplepsyres.2020.106370. PMID: 32516743.

22. Arksey H, O'Malley L. Scoping studies: Towards a methodological framework. Int J Soc Res Methodol Theory Pract. 2005;8(1):19–32. https://doi.org/10.1080/1364557032000119616. PMID: 20854677.

23. Peters MDJ, Godfrey C, McInerney P, Munn Z, Tricco AC, Khalil, H. Chapter 11: Scoping Reviews (2020 version). In: Aromataris E, Munn Z (Editors). JBI Manual for Evidence Synthesis, JBI, 2020. https://doi.org/10.46658/JBIMES-20-12.

24. Joanna Briggs Institute. "The Joanna Briggs Institute reviewers' manual 2015: Methodology for JBI scoping reviews." *Adelaide: The Joanna Briggs Institute* (2015): 6-22.

25. Shamseer L, Moher D, Clarke M, Ghersi D, Liberati A, Petticrew M, et al. Preferred reporting items for systematic review and meta-analysis protocols (PRISMA-P) 2015: elaboration and explanation. Bmj. 2015; 349. https://doi.org/10.1136/bmj.g7647 PMID: 25555855.

26. Tricco AC, Lillie E, Zarin W, O'Brien KK, Colquhoun H, Levac D, et al. PRISMA Extension for Scoping Reviews (PRISMA-ScR): Checklist and Explanation. Ann Intern Med. 2018; 169(7):467–73. https://doi.org/10.7326/M18-0850. PMID: 30178033.

27. World Health Organization. Poluição do ar e saúde infantil: prescrevendo ar puro. 2018.

28. World Health Organization (WHO). Ageing and Health Technical Report. A glossary of terms. 2004;5:111.

29. Zhu N, Zhang D, Wang W, et al. A novel coronavirus from patients with pneumonia in China, 2019. N Engl J Med 2020;382:727–33. https://doi.org/10.1056/NEJMoa2001017. PMID: 31978945.

30. World Health Organization. Coronavirus disease (COVID-19) outbreak. Geneva: WHO, 2020.

31. Lopes-Júnior LC, Bomfim EO, Nascimento LC, Nunes MD, Pereira-da-Silva G, Lima RA. Non-pharmacological interventions to manage fatigue and psychological stress in children and adolescents with cancer: an integrative review. Eur J Cancer Care (Engl). 2016 Nov;25(6):921-935. https://doi.org/10.1111/ecc.12381. PMID: 26374619.

32. Lopes-Júnior LC, Bomfim E, Olson K, Neves ET, Silveira DSC, Nunes MDR, et al. Effectiveness of hospital clowns for symptom management in paediatrics: systematic review of randomised and non-randomised controlled trials. BMJ. 2020 Dec 16;371:m4290. doi: https://doi.org/10.1136/bmj.m4290. PMID: 33328164.

33. Lopes-Júnior LC, Rosa GS, Pessanha RM, Schuab SIPC, Nunes KZ, Amorim MHC. Efficacy of the complementary therapies in the management of cancer pain in palliative care: a systematic review. Rev Lat Am Enfermagem. 2020; 28:e3377. https://doi.org/10.1590/1518-8345.4213.3377. PMID: 33027406.

34. Lopes-Júnior LC, Rosa MADRP, Lima RAG. Psychological and Psychiatric Outcomes Following PICU Admission: a systematic review of cohort studies. Pediatr Crit Care Med. 2018; 19(1):e58-e67. https://doi.org/doi:10.1097/PCC.0000000000001390 PMID: 29189670

35. Lopes-Júnior LC, Bomfim E, Silveira DSCD, Pessanha RM, Schuab SIPC, Lima RAG. Effectiveness of mass testing for control of COVID-19: a systematic review protocol. BMJ Open. 2020;10(8):e040413. http://dx.doi.org/10.1136/bmjopen-2020-040413. PMID: 32847926

36. Silva Junior FJGD, Sales JCES, Monteiro CFS, Costa APC, Campos LRB, Miranda PIG, et al. Impact of COVID-19 pandemic on mental health of young people and adults: a systematic review protocol of observational studies. BMJ Open. 2020;10(7): e039426. http://dx.doi.org/10.1136/bmjopen-2020-039426 PMID: 32611746

Click here to access/download
Supporting Information
PRISMA-P 2015 checklist PLOS ONE 7.8.2021.docx
