# Supplementary material for: Telehealth in outpatient care for children and adolescents with chronic conditions during the COVID-19 pandemic: A scoping review protocol
Source: PLoS One. 2022 Jun 10;17(6):e0269821. doi: 10.1371/journal.pone.0269821 (PMC9187091; doi:10.1371/journal.pone.0269821)
Supplement: Attachment — Submitted filename: Response to Reviewers.docx [file pone.0269821.s003.docx]

**Response letter to the Editor and reviewers**

Ribeirão Preto, SP, Brazil, March 16^th^ , 2022

Coments of Academic Editor, Rizaldy Taslim Pinzon

PONE-D-21-25612

Telehealth in outpatient care for children and adolescents with chronic conditions during the COVID-19 pandemic: a scoping review protocol

PLOS ONE

Dear Dr. Regina Aparecida Garcia Lima

Thank you for submitting your manuscript to PLOS ONE. After careful consideration, we feel that it has merit but does not fully meet PLOS ONE’s publication criteria as it currently stands. Therefore, we invite you to submit a revised version of the manuscript that addresses the points raised during the review process.

**Response:** *Dear Dr.* Rizaldy Taslim Pinzon*,*

*We would like to thank you for the opportunity to review the manuscript after the reviewers' suggestions and recommendations.*

*All points were addressed and/or clarified in this new version. In addition, we responded item by item to the questions raised by the reviewers in this letter.*

-If you would like to make changes to your financial disclosure, please include your updated statement in your cover letter. Guidelines for resubmitting your figure files are available below the reviewer comments at the end of this letter.

**Response:** It is not necessary. Thanks!

**Journal Requirements:**

Please review your reference list to ensure that it is complete and correct. If you have cited papers that have been retracted, please include the rationale for doing so in the manuscript text, or remove these references and replace them with relevant current references. Any changes to the reference list should be mentioned in the rebuttal letter that accompanies your revised manuscript. If you need to cite a retracted article, indicate the article’s retracted status in the References list and also include a citation and full reference for the retraction notice.

**Response:** We have carefully reviewed the reference list and we ensure that it is complete and correct. Also, we have no papers retracted cited in this manuscript.

**Reviewers' comments:**

Reviewer's Responses to Questions

**Comments to the Author**

1. Does the manuscript provide a valid rationale for the proposed study, with clearly identified and justified research questions?

The research question outlined is expected to address a valid academic problem or topic and contribute to the base of knowledge in the field.

Reviewer #1: Yes

2. Is the protocol technically sound and planned in a manner that will lead to a meaningful outcome and allow testing the stated hypotheses?

The manuscript should describe the methods in sufficient detail to prevent undisclosed flexibility in the experimental procedure or analysis pipeline, including sufficient outcome-neutral conditions (e.g. necessary controls, absence of floor or ceiling effects) to test the proposed hypotheses and a statistical power analysis where applicable. As there may be aspects of the methodology and analysis which can only be refined once the work is undertaken, authors should outline potential assumptions and explicitly describe what aspects of the proposed analyses, if any, are exploratory.

Reviewer #1: Yes

3. Is the methodology feasible and described in sufficient detail to allow the work to be replicable?

Reviewer #1: Yes

4. Have the authors described where all data underlying the findings will be made available when the study is complete?

The [PLOS Data policy](https://journals.plos.org/plosone/s/materials-and-software-sharing) requires authors to make all data underlying the findings described in their manuscript fully available without restriction, with rare exception, at the time of publication. The data should be provided as part of the manuscript or its supporting information, or deposited to a public repository. For example, in addition to summary statistics, the data points behind means, medians and variance measures should be available. If there are restrictions on publicly sharing data—e.g. participant privacy or use of data from a third party—those must be specified.

Reviewer #1: Yes

5. Is the manuscript presented in an intelligible fashion and written in standard English?

*PLOS ONE* does not copyedit accepted manuscripts, so the language in submitted articles must be clear, correct, and unambiguous. Any typographical or grammatical errors should be corrected at revision, so please note any specific errors here.

Reviewer #1: Yes

6. Review Comments to the Author

Please use the space provided to explain your answers to the questions above and, if applicable, provide comments about issues authors must address before this protocol can be accepted for publication. You may also include additional comments for the author, including concerns about research or publication ethics.

You may also provide optional suggestions and comments to authors that they might find helpful in planning their study.

Reviewer #1:

The protocol titled Telehealth in outpatient care for children and adolescents with chronic conditionsduring the COVID-19 pandemic: a scoping review protocol seems to be good study and authors will try to find out ways to follow up chronic cases through telehealth after searching published literatures .

***Response:*** *Thank you so much for you positive feedback.*

- Hence in introduction I have come comments.
- Line 104: Spell check of highlighted word

**Response:** Done! Thanks for your careful review.

Line 110-112: Can be omitted

**Response:** We have omitted the sentence as per suggested. Thanks.

While revising your submission, please upload your figure files to the Preflight Analysis and Conversion Engine (PACE) digital diagnostic tool, <https://pacev2.apexcovantage.com/>. PACE helps ensure that figures meet PLOS requirements. To use PACE, you must first register as a user. Registration is free. Then, login and navigate to the UPLOAD tab, where you will find detailed instructions on how to use the tool. If you encounter any issues or have any questions when using PACE, please email PLOS at [figures@plos.org](mailto:figures@plos.org). Please note that Supporting Information files do not need this step.

**Response:** Not applicable

The authors
