# Supplementary material for: Telehealth in outpatient care for children and adolescents with chronic conditions during the COVID-19 pandemic: A scoping review protocol
Source: PLoS One. 2022 Jun 10;17(6):e0269821. doi: 10.1371/journal.pone.0269821 (PMC9187091; doi:10.1371/journal.pone.0269821)
Supplement: Attachment — Submitted filename: Response to Reviewers.docx [file pone.0269821.s004.docx]

**Response to Reviewers**

Ribeirão Preto, SP, Brazil, May 5th, 2022

*Minor revision*

PONE-D-21-25612R1

Telehealth in outpatient care for children and adolescents with chronic conditions during the COVID-19 pandemic: a scoping review protocol

Dear Dr. Regina Aparecida Garcia Lima
Thank you for submitting your manuscript to PLOS ONE. After careful consideration, we feel that it has merit but does not fully meet PLOS ONE’s publication criteria as it currently stands. Therefore, we invite you to submit a revised version of the manuscript that addresses the points raised during the review process.

There are some concern about methodological flaws in your study. Please make the revision based on reviewers suggestion and comments.

**Response:** Dear Dr. Rizaldy Taslim Pinzon,

Thank you for the opportunity to review the manuscript after the reviewers' suggestions and recommendations.

All points were addressed and/or clarified in this new version. In addition, we responded item by item to the questions raised by the reviewers in this letter.

Please review your reference list to ensure that it is complete and correct. If you have cited papers that have been retracted, please include the rationale for doing so in the manuscript text, or remove these references and replace them with relevant current references. Any changes to the reference list should be mentioned in the rebuttal letter that accompanies your revised manuscript. If you need to cite a retracted article, indicate the article’s retracted status in the References list and also include a citation and full reference for the retraction notice.

**Response:** We have carefully reviewed the reference list and we ensure that it is complete and correct. Also, we have no papers retracted cited in this manuscript.

**Reviewers' comments:**

Reviewer's Responses to Questions

**Comments to the Author**

1. Does the manuscript provide a valid rationale for the proposed study, with clearly identified and justified research questions?
The research question outlined is expected to address a valid academic problem or topic and contribute to the base of knowledge in the field.

Reviewer #2: Yes

2. Is the protocol technically sound and planned in a manner that will lead to a meaningful outcome and allow testing the stated hypotheses?
The manuscript should describe the methods in sufficient detail to prevent undisclosed flexibility in the experimental procedure or analysis pipeline, including sufficient outcome-neutral conditions (e.g. necessary controls, absence of floor or ceiling effects) to test the proposed hypotheses and a statistical power analysis where applicable. As there may be aspects of the methodology and analysis which can only be refined once the work is undertaken, authors should outline potential assumptions and explicitly describe what aspects of the proposed analyses, if any, are exploratory.

Reviewer #2: Yes

3. Is the methodology feasible and described in sufficient detail to allow the work to be replicable?

Reviewer #2: No

4. Have the authors described where all data underlying the findings will be made available when the study is complete?
The [PLOS Data policy](https://journals.plos.org/plosone/s/materials-and-software-sharing) requires authors to make all data underlying the findings described in their manuscript fully available without restriction, with rare exception, at the time of publication. The data should be provided as part of the manuscript or its supporting information, or deposited to a public repository. For example, in addition to summary statistics, the data points behind means, medians and variance measures should be available. If there are restrictions on publicly sharing data—e.g. participant privacy or use of data from a third party—those must be specified.

Reviewer #2: Yes

5. Is the manuscript presented in an intelligible fashion and written in standard English?
*PLOS ONE* does not copyedit accepted manuscripts, so the language in submitted articles must be clear, correct, and unambiguous. Any typographical or grammatical errors should be corrected at revision, so please note any specific errors here.

Reviewer #2: Yes

6. Review Comments to the Author
Please use the space provided to explain your answers to the questions above and, if applicable, provide comments about issues authors must address before this protocol can be accepted for publication. You may also include additional comments for the author, including concerns about research or publication ethics.

You may also provide optional suggestions and comments to authors that they might find helpful in planning their study.

**Reviewer #2:**

In the context of the COVID-19 pandemic, this is certainly an interesting project and well targeted. The title is well aligned with the objectives.

**Response:** Thank you so much for your comments.

However, the methods need an extensive revision. A librarian support is needed in order to render the search strategy more robust.

**Response:** A librarian was consulted for checking the search strategy. We added in this version the complete strategy revised by the librarian of the Federal University of Espírito Santo, respecting the controlled descriptors (subject indexers in each database) and also combining with the synonyms and keywords in order to make the search strategy broad and robust.

**Introduction:**
P3 L110 What is clinical weaknesses? Details or more clarifications are needed.

**Response:** We edited this sentence to make it clearer. “Children and adolescents with chronic conditions demand high hospital readmission rates,……”

P3 L130 -P4 L131-133What this is an added value? Use in what context???

**Response:** We edited this sentence to make it clearer. “Telehealth is being used in several countries worldwide [15–21], especially in non-emergency/routine care and in cases where services do not require direct patient-provider interaction such as providing psychological services, mainly in the current scenario of COVID-19 pandemic in order to nullify the barriers imposed by the COVID-19 pandemic. Additionally, telehealth can become a basic need for the general population, health care providers, and patients with COVID-19, mainly when people are in quarantine, enabling patients in real time through contact with health care provider for advice on their health problems”.
 **Methods**
P4, L151-155 The reference of Tricco et al 2018 suffices.

**Response:** OK. Done. Thanks!

P4 L163-4 what is the added value of this classification? In case it is worth, please elaborate.

**Response:** OK. We removed the classification.

P5 L180-1 the definition of outpatient care is very simplistic and may limit the scope of your findings.

**Response:** OK. Rewriten.

P5 L194 certainly assumed. So, no need to mention this “using the Boolean terms AND/OR”.

**Response:** OK. Done.

Table 1 based on the study title and the objectives; some essential key concepts are missing. They are outpatient care and chronic conditions. They are totally ignored.

**Response:** OK. We have added to the search strategy. Many Thanks!

Table 1 Could authors explain why [All fields] is used. Because it is classical to capture references through titles and abstract. Otherwise, this will lead to incredible volume of « noise ». Please elaborate.

**Response:** OK. We reworded it with the help of the librarian.

Table 1 Do authors sought a support from librarians? This expertise is essential. #1 AND #2 AND #3 needs AND outpatient…..AND chronic….

**Response:** OK. We reworded it with the help of the librarian.

The type of design to be included or excluded is not stated.

**Response:** We have added “study design”. Thanks.

P5 L222 is it a typo «2021».

**Response:** Sure. Thank you. Edited for “2022”.

P5, L245-6 Please remove this as there is no added value.

**Response:** Ok. Removed.

P8 L259 In what this is a limitation since you don’t what will be found.

**Response:** Ok. Removed.

7. PLOS authors have the option to publish the peer review history of their article ([what does this mean?](https://journals.plos.org/plosone/s/editorial-and-peer-review-process#loc-peer-review-history)). If published, this will include your full peer review and any attached files.

If you choose “no”, your identity will remain anonymous but your review may still be made public.

**Do you want your identity to be public for this peer review?** For information about this choice, including consent withdrawal, please see our [Privacy Policy](https://www.plos.org/privacy-policy).

Reviewer #2: No

[NOTE: If reviewer comments were submitted as an attachment file, they will be attached to this email and accessible via the submission site. Please log into your account, locate the manuscript record, and check for the action link "View Attachments". If this link does not appear, there are no attachment files.]
While revising your submission, please upload your figure files to the Preflight Analysis and Conversion Engine (PACE) digital diagnostic tool, <https://pacev2.apexcovantage.com/>. PACE helps ensure that figures meet PLOS requirements. To use PACE, you must first register as a user. Registration is free. Then, login and navigate to the UPLOAD tab, where you will find detailed instructions on how to use the tool. If you encounter any issues or have any questions when using PACE, please email PLOS at [figures@plos.org](mailto:figures@plos.org). Please note that Supporting Information files do not need this step.

*In compliance with data protection regulations, you may request that we remove your personal registration details at any time.*  [*(Remove my information/details)*](https://www.editorialmanager.com/pone/login.asp?a=r)*. Please contact the publication office if you have any questions.*
